# Acceptability and Implementation Considerations for Community Pharmacy–Based Delivery of Pre-Exposure Prophylaxis and Antiretroviral Therapy in Dar es Salaam: A Sequential Explanatory Mixed-Methods Design

**DOI:** 10.21203/rs.3.rs-9530938/v1

**Published:** 2026-05-12

**Authors:** George Msema Bwire, Annabel Itaeli, Japhet Killewo, Christopher R. Sudfeld

**Affiliations:** Muhimbili University of Health and Allied Sciences; Muhimbili University of Health and Allied Sciences; Muhimbili University of Health and Allied Sciences; Harvard T.H. Chan School of Public Health

**Keywords:** Community pharmacies, HIV service delivery, pre-exposure prophylaxis, Antiretroviral therapy, Tanzania

## Abstract

**Background:**

Community pharmacies (CPs) may provide a decentralized platform for refills of pre-exposure prophylaxis (PrEP) and antiretroviral therapy (ART). However, evidence on their acceptability and implementation requirements remains limited. This study assessed the acceptability of using CP for PrEP and ART refills and explored stakeholder perspectives on feasibility to inform potential scale-up in urban Tanzania.

**Methods:**

A sequential explanatory mixed-methods design study of community pharmacies was conducted in Dar es Salaam, Tanzania. Quantitative surveys were first conducted among women engaged in transactional sex and people living with HIV (PLHIV) to assess the acceptability and feasibility of obtaining PrEP or refilling ART through CPs. These findings then informed qualitative data collection, which included in-depth interviews with women engaged in transactional sex (TS), pharmacists, pharmacy owners, and key informants such as policymakers, as well as focus-group discussions with PLHIV. Qualitative data were analyzed thematically to contextualize and explain the quantitative results.

**Results:**

Nearly 74.2% of the 93 women engaged in TS expressed willingness to receive PrEP from CPs. Among PLHIV, 66.0% of the 341 participants reported willingness to refill ART at community pharmacies. Across both groups, major concerns included stigma related to HIV status or perceived sexual behavior and potential user fees. Qualitative findings (N = 27) highlighted perceived advantages of pharmacies such as proximity, shorter waiting times, flexible opening hours, and the ability to obtain services discreetly within routine care settings. However, participants emphasized that acceptability would depend on assured confidentiality, free or affordable treatment, private consultation spaces, and specialized HIV training for pharmacy staff. Stakeholders further identified regulatory and financing uncertainties, infrastructure constraints, workforce shortages, medication misuse risks, and the need for digital health record systems as implementation challenges.

**Conclusions:**

CPs were perceived as promising complementary platforms for PrEP delivery, particularly to improve prevention access among high-risk populations, and for ART refills mainly among stable clients. However, successful integration into national HIV programs will require robust regulatory frameworks, sustainable financing, provider training, and systems to safeguard confidentiality and continuity of care. Implementation research is needed to evaluate feasibility, impact, and safety.

## Background

HIV remains a major global public health challenge, with an estimated 39 million people living with HIV worldwide and approximately 1.3 million new infections occurring annually despite major advances in treatment and prevention [1]. Although large-scale antiretroviral therapy (ART) programs have reduced AIDS-related mortality by more than 60% since 2004, persistent HIV transmission underscores the need to strengthen biomedical prevention strategies alongside treatment scale-up. Global HIV responses increasingly emphasize antiretroviral-based interventions, including pre-exposure prophylaxis (PrEP) and post-exposure prophylaxis (PEP), delivered as part of combination prevention approaches [2]. Oral PrEP can reduce sexual HIV acquisition by up to 99%, while timely PEP is highly effective following sexual or parenteral exposures [3].

Sub-Saharan Africa continues to experience the greatest burden of HIV, driven by structural barriers such as stigma, limited health system capacity, and inequitable access to services [4]. In Tanzania, approximately 1.5 million adults are living with HIV, with an estimated annual incidence of 0.18%, corresponding to around 60,000 new infections [5]. On the other hand, Tanzania has made substantial progress in ART delivery, with 97.9% of diagnosed individuals receiving treatment and 94.3% of those on ART achieving viral suppression [6]. However, service delivery remains concentrated in public-sector facilities, creating challenges related to long waiting times, transportation costs, and stigma for clients [7]. These same barriers constrain access to PrEP, which is primarily offered through public facilities and targeted programs, leaving many individuals, particularly those in underserved settings, without convenient prevention options [8], [9].

Differentiated service delivery models have been promoted to decentralize HIV treatment and prevention and improve client-centered care [10], [11], [12]. Community pharmacy represents a promising platform for expanding access to PrEP and ART refills because of their wide reach, extended operating hours, and perceived privacy. In Tanzania, these outlets serve nearly 70% of rural and semi-urban populations, and more than 6,000 are registered nationwide [13]. Expanding pharmacy-based delivery of PrEP and ART services could improve adherence and retention, reduce pressure on overburdened facilities, and strengthen Tanzania’s combination prevention strategy through task sharing with pharmacists [14]. Leveraging community pharmacies for ART and PrEP delivery has the potential to reduce structural and social barriers, expand reach to underserved populations, and strengthen Tanzania’s combination prevention strategy. This study assessed the acceptability of community pharmacy-based PrEP delivery and ART refills and explored stakeholder perspectives on the feasibility to inform potential scale-up of decentralized HIV prevention and treatment services.

## Methods

### Study design and setting

This study employed a sequential explanatory mixed-methods design, in which quantitative surveys were first conducted among women engaged in transactional sex and people living with HIV (PLHIV) to assess acceptability to obtain PrEP or refill ART through community pharmacies in the Dar es Salaam Region of Tanzania beginning in September 2025,, followed by qualitative methods to explore experiences, perceptions, and contextual factors influencing feasibility and implementation. Dar es Salaam was selected as the study setting because it is Tanzania’s largest urban center and commercial hub, with rapid urbanization and a high concentration of health service delivery points. According to national census data, the region has a population exceeding five million, representing the largest share of Tanzania’s total population of more than 60 million [15]. Tanzania’s HIV epidemic is generalized, with an estimated adult HIV prevalence of approximately 4.4%, while Dar es Salaam has a comparable prevalence of about 4.2% [5]. Evidence from Dar es Salaam indicates that HIV prevalence among females engaged in transactional sex is substantially higher than in the general population, with estimates of approximately 15% [16]. In addition, an estimated 6,000 females engaged in transactional sex reside in the Dar es Salaam Region [17].

### Study population

The primary study populations comprised females engaged in transactional sex, defined as the exchange of sexual services for money, goods, or other material benefits, selected to represent individuals at high risk of HIV acquisition, as well as PLHIV. Eligible females engaged in transactional sex were aged 18 years or older, resided in Dar es Salaam, and reported exchanging sex for money, goods, or material support within the past six months; those unable to provide informed consent were excluded. PLHIV were eligible if they were aged 18 years or older, receiving ART at selected HIV clinics in Dar es Salaam, and willing to participate.

[16][17]Participants engaged in transactional sex were included in both the cross-sectional quantitative survey and qualitative in-depth interviews (IDIs). PLHIV participated in the quantitative component and focus group discussions (FGDs). To complement these perspectives, community pharmacists, pharmacy owners, and institutional stakeholders involved in HIV prevention, pharmaceutical service delivery, and regulation were included through key informant interviews (KIIs).

For females engaged in transactional sex, given the hidden and stigmatized nature of this population and the lack of a formal sampling frame, respondent-driven sampling was used for both the survey and interviews. Initial participants (“seeds”) were identified through community networks and recruited peers until the quantitative sample size and qualitative data saturation were reached.

PLHIV were recruited from HIV clinics using a systematic sampling approach. After completion of the quantitative survey, females engaged in transactional sex and PLHIV were subsequently invited to participate in IDIs and FGDs, respectively. For stakeholder perspectives, IDIs were conducted with community pharmacists and pharmacy owners. Purposive sampling was used to recruit these participants, along with other institutional stakeholders involved in HIV prevention, pharmaceutical service delivery, and regulation [18]. Selection of participants was based on professional roles, experience, and relevance to the implementation of community pharmacy–based PrEP and ART refill services [19].

### Sample size estimation

Quantitative sample sizes were calculated using the standard cross-sectional formula $n=Z^{2}p(1-p)∕E^{2}$ [18]. For females engaged in transactional sex, a 95% confidence level (Z = 1.96), a margin of error (E) of 10%, and an estimated population of approximately 6,000 females involved in transactional sex in Dar es Salaam [17], were assumed, yielding a minimum required sample size of 90 participants and a minimum of 340 for PLHIV with a margin of error of 5.5%. For the qualitative component, purposive sampling was used to recruit participants based on their roles, experience, and relevance to community pharmacy–based HIV service delivery, including women engaged in transactional sex, PLHIV, community pharmacists and pharmacy owners in Dar es Salaam, and national and regional policymakers and regulators. The qualitative sample size was guided by the principle of data saturation, with recruitment continuing until no new themes emerged.

### Data collection process

The quantitative questionnaire was developed based on Theoretical Framework of Acceptability, the acceptability–appropriateness–feasibility framework acceptability [19], [20], and was further informed by constructs adapted from prior research evidence [10], [12], [21], [22], [23]. These frameworks and research evidence guided the inclusion of domains assessing perceived acceptability and feasibility to use pharmacy-based ART and PrEP refill services, and the perceived practicality of implementing such services within community pharmacy settings.

Separate structured questionnaires were administered to females engaged in transactional sex and to PLHIV. The PrEP questionnaire, given to females engaged in transactional sex, included items on PrEP awareness, willingness to obtain PrEP from community pharmacies, preferred service characteristics, perceived benefits, concerns such as stigma and cost, and support needs. The ART questionnaire, administered to PLHIV receiving care at HIV clinics, assessed willingness to refill ART at community pharmacies, prior awareness of pharmacy-based ART services, perceived advantages and barriers, service preferences, and desired additional pharmacy-based services (**Supplementary file 1**).

The qualitative component employed semi-structured interviews to explore experiences and contextual factors influencing the acceptability and feasibility of community pharmacy–based PrEP refill services. Separate interview guides were developed for females involved in transactional sex, PLHIV and for key stakeholders (including community pharmacists, pharmacy owners, and institutional representatives).

Guides for females engaged in transactional sex and PLHIV explored experiences accessing HIV services, perceptions of pharmacy based PrEP and ART delivery, stigma related concerns, and conditions influencing acceptability, while stakeholder guides included additional probes on regulatory, operational, workforce, infrastructure, financing, and data system considerations. For stakeholders, additional probes explored regulatory, operational, and system-level considerations relevant to pharmacy-based ART and PrEP delivery.

The tools were pre-tested with a small sample of participants from a non-study site to assess clarity, comprehension, and acceptability. Based on feedback, minor revisions were made prior to full deployment, including rewording of selected items to improve clarity, simplifying technical terminology related to ART and PrEP, adjusting the sequence of questions to improve flow, and adding brief explanations to Likert-scale response options to enhance participant understanding. Quantitative data were collected using a structured questionnaire administered through the online KoboToolbox platform (Harvard Humanitarian Initiative, Cambridge, MA, USA). Qualitative data were collected through interviews conducted in Kiswahili, the local language, which were audio-recorded and later transcribed verbatim for analysis.

### Data analysis

Data collected using the Kobo Toolbox platform (Harvard Humanitarian Initiative, Cambridge, MA, USA) were exported to Microsoft Excel (Microsoft Corporation, Redmond, WA, USA) for data cleaning and management. Descriptive statistics were employed to summarize all study variables using appropriate measures of central tendency and variability, including means (standard deviation), medians (interquartile range: 25% – 75%), standard deviations, frequencies, and proportions. Variables summarized included sociodemographic characteristics, HIV related service use characteristics, awareness and preferences regarding pharmacy-based PrEP and ART services, and perceived benefits and concerns. Willingness was measured using a 5-point Likert scale and dichotomized as willing (strongly agree/agree) or not willing (neutral/disagree/strongly disagree) [24]. Chi-square tests were used to assess associations between willingness to obtain PrEP from a community pharmacy and categorical independent variables. These analyses correspond to the assessment of factors associated with willingness presented in Table 1. Quantitative data were analyzed using R statistical software, with statistical significance set at p < 0.05 [25].

For the qualitative component, audio recordings were transcribed verbatim and translated into English before analysis. The qualitative methods were reported in accordance with the Consolidated Criteria for Reporting Qualitative Research (COREQ) guidelines [26]. Semi structured interview and focus group guides were developed based on the Theoretical Framework of Acceptability and the acceptability, appropriateness, and feasibility framework, as well as findings from the initial quantitative phase. Guides were reviewed by the multidisciplinary research team and piloted at a non study site prior to data collection.

Interviews and focus group discussions were conducted in Kiswahili by trained qualitative researchers with prior experience in HIV research and community based data collection. Field notes were taken during and immediately after each interview or discussion to document non verbal cues, contextual observations, and preliminary analytic reflections. Interviews and discussions were conducted in private settings to ensure confidentiality.

Thematic analysis was conducted through systematic coding, categorization, and iterative refinement of themes, with illustrative quotations selected to exemplify key findings [25]. Transcripts were independently reviewed by at least two members of the research team to enhance analytic rigor. An initial coding framework was developed deductively from the interview guides and relevant implementation frameworks, and further refined inductively as new themes emerged from the data. Coding discrepancies were discussed and resolved through consensus. Data management and coding were conducted using qualitative analysis NVivo software [27]. Recruitment continued until thematic saturation was reached.

## Results

### Quantitative findings

#### PrEP refill through community pharmacies

The quantitative survey included 93 females engaged in transactional sex. Most participants were aged ≥ 28 years (44.1%), and nearly half initiated transactional sex between 23 and 27 years (46.2%). The majority had 2–5 years of sex work experience (44.1%) and reported ≥ 3 clients per day (57.0%). Most were not married (95.7%), 57.0% had children, 51.6% had completed secondary education (21.6% diploma or higher), and 73.1% were not formally employed. In addition, 67.7% reported consistent condom use and 54.8% had experienced violence or coercion. Overall, 74.2% of participants expressed willingness to receive PrEP at a community pharmacy. Willingness did not differ significantly across most sociodemographic or work-related characteristics (all p > 0.05), except for education level, where higher education was associated with greater willingness (p = 0.047). Table 1.

#### Participant preferences and concerns about PrEP services

Most participants obtained PrEP information from health workers (60.2%). Pharmacies were the preferred PrEP access point for 62.4%, and 80.6% favored refill intervals of two months or longer. Interest in long-acting injectable PrEP was high (77.4%), while most had no preference regarding provider sex (89.2%). Major concerns about pharmacy-based PrEP included fear of being judged (98.9%), cost (58.1%), and stigma or discrimination (49.5%). Convenience was noted by 50.5% of participants, whereas fewer reported worries about medication quality (14.0%) or stockouts (6.5%). Nearly all respondents preferred counselling or support services (98.9%), and 45.2% desired peer support (Table 2).

#### ART refill through community pharmacies

Among the 341 PLHIV included in the analysis, 225 (66.0%) reported willingness to refill ART at a community pharmacy. Willingness differed significantly by sex and prior awareness of pharmacy-based ART services: men were more likely than women to report willingness (p = 0.1). No other demographic, socioeconomic, clinical, or access-related characteristics, including age, marital status, education, employment status, time since HIV diagnosis or ART initiation, clinic travel time, waiting time, medication support systems, or prior refill challenges were significantly associated with willingness (all p > 0.05) Table 3.

#### Awareness, perceived benefits, and concerns regarding pharmacy-based ART services

A total of 341 PLHIV completed questions on awareness, perceived benefits, concerns, and preferences regarding community pharmacy–based ART services. Only 14.7% had previously heard of pharmacy-based ART services, and none had ever received ART from a private pharmacy. Nevertheless, respondents frequently identified practical advantages, including shorter waiting times (63.6%), reduced transportation costs (62.8%), and proximity to home (52.8%). Concerns were common, particularly fear of stigma (74.8%) and confidentiality (61.3%), while approximately one-quarter cited high cost or worries about medication quality. Apprehension about pharmacist expertise (7.0%) and record-keeping (1.2%) were uncommon. Acceptance of long-acting injectable ART was high (91.8%). Interest in additional pharmacy-based services was also substantial, especially general health screening (60.7%) and adherence counseling (57.8%) Table 4.

### Qualitative findings

#### Overview of qualitative participants

In total 27 participants were interviewed, the qualitative dataset comprised 7 IDIs with women engaged in transactional sex, 6 IDIs with pharmacists and pharmacy owners, 2 KIIs with policymakers and regulators, and 3 FGDs with PLHIV. Women engaged in transactional sex were interviewed to assess the acceptability of pharmacy-based PrEP delivery; PLHIV participated in FGDs to explore perceptions of ART refills in community pharmacies; pharmacists and pharmacy owners were interviewed to identify operational and infrastructural requirements for implementation; and policymakers were interviewed to examine the regulatory and policy context governing community-based HIV service delivery.

Across all qualitative participants, ages ranged from 22 to 52 years, with an overall median age of 32 years (IQR: 27–38). Among women engaged in transactional sex (n = 7), the mean age was 26.7 years (SD 4.2); all were female, the median number of children was one (range 0–1), and three reported current PrEP use while four had never used PrEP. People living with HIV who participated in FGDs (n = 12) had a mean age of 36.1 years (SD 8.4); ten were female and two males, with a median time on ART of five years (IQR: 3–8). Five reported community pharmacies as their first point of contact for care, while seven primarily used health facilities or hospitals. Pharmacists and health-system stakeholders interviewed through IDIs (n = 7) had a mean age of 32.0 years (SD 3.3); five were male and two females, and the median duration of professional experience was six years (IQR: 5–9), with two reporting direct involvement in HIV service delivery. Key informants and policymakers (n = 2) had a mean age of 44 years; one was male and one female, and their median professional experience was seven years (**Supplementary file 2)**.

#### Acceptability of PrEP refills at community pharmacies

Analysis of interviews with women engaged in transactional sex identified six major themes and eighteen sub-themes related to the acceptability of PrEP delivery through community pharmacies. Overall, participants largely viewed community pharmacies as an acceptable and often preferable setting for PrEP provision compared with hospitals. Pharmacies were valued for their accessibility, shorter waiting times, discretion, and lower perceived costs, which were seen as reducing fears of stigma and unwanted disclosure.

However, acceptability was highly conditional on several safeguards. Participants highlighted the need for improved training of pharmacy staff, availability of private counselling spaces, clear and targeted health education for sex workers, and deliberate efforts to dispel misconceptions surrounding PrEP. In the absence of these measures, mistrust in providers, fear of judgment, and persistent misinformation were perceived as likely to undermine uptake and limit the effectiveness of pharmacy-based PrEP delivery models (Table 5).

#### Acceptability of ART refills at community pharmacies

Analysis of the focus-group discussions with PLHIV identified eight major themes and twenty-two sub-themes regarding the acceptability of ART refills at community pharmacies among people living with HIV. Participants highlighted potential advantages such as reduced travel distance, shorter waiting times, flexible pharmacy opening hours, and access during emergencies. However, strong concerns emerged around stigma, breaches of confidentiality, possible user fees, and the perceived lack of HIV-specific expertise among pharmacy staff. Hospitals were widely trusted for providing counselling, psychosocial support, and clinical monitoring.

Overall, willingness to use community pharmacies was mixed. Participants emphasized that acceptability would depend on ART remaining free of charge, the availability of private and discreet dispensing areas, and the presence of specially trained personnel capable of maintaining confidentiality and providing appropriate clinical support. Without these safeguards, most participants preferred to continue obtaining ART through hospital-based services (Table 6).

#### Barriers and facilitators to community pharmacy delivery of ART and PrEP

Across the analysis, nine major themes comprising multiple interrelated sub-themes were identified (Table 7). Overall, participants perceived community pharmacies as promising platforms for decentralized ART and PrEP delivery, particularly because of their accessibility and potential to reduce stigma. However, this optimism was consistently balanced by concerns related to operational readiness, especially issues surrounding medicine misuse, incomplete dosing, and the need for strong counselling and monitoring systems.

Structural and organizational constraints also featured prominently, with recurring sub-themes relating to limited staffing, heavy workloads, and inadequate private spaces for confidential consultations. At the system level, participants repeatedly pointed to regulatory oversight, financing arrangements, and interoperable digital platforms as prerequisites for safe implementation. Collectively, these patterns suggest that while the policy environment is generally supportive, successful scale-up will depend on addressing a small set of foundational challenges related to workforce capacity, infrastructure, financing, and data integration.

## Discussion

This mixed methods study demonstrates substantial but conditional support for decentralizing HIV prevention and treatment services to community pharmacies. Quantitatively, a large proportion of women engaged in transactional sex were willing to obtain PrEP through pharmacies, while a smaller but still notable proportion of people living with HIV were willing to refill ART in these settings. Qualitative findings helped explain these patterns by showing that pharmacies were widely perceived as convenient and accessible because of their proximity, shorter waiting times, and flexible hours, but concerns related to stigma, confidentiality, potential costs, and provider competence strongly shaped acceptability.

Participants emphasized that pharmacy-based HIV service delivery would only be acceptable if key safeguards were in place, including trained staff, private consultation spaces, reliable supply systems, and maintenance of free or affordable treatment. Stakeholders further highlighted important implementation challenges, such as infrastructure limitations, workforce capacity constraints, regulatory and financing uncertainties, risks of medication misuse, and the need for interoperable digital health records. Together, these findings suggest that while community pharmacies are viewed as promising complementary platforms for HIV service delivery, successful integration will depend on addressing both individual level acceptability concerns and broader health system readiness factors.

Among women at high risk of HIV infection, nearly three-quarters expressed willingness to receive PrEP from community pharmacies, and pharmacies were the most preferred access point for PrEP services. This aligns with qualitative findings in which women consistently emphasized proximity, shorter waiting times, and reduced visibility compared with hospital-based HIV clinics. These features are particularly salient for populations facing stigma and time constraints, suggesting that pharmacy-based models could lower structural barriers to PrEP continuation and refill adherence [10], [28].Notably, willingness to receive PrEP at pharmacies did not vary by most sociodemographic or work-related characteristics, indicating broad acceptability across subgroups of sex workers. Education level was the only factor associated with willingness, with higher education linked to greater acceptance, pointing to the potential role of health literacy in shaping confidence in decentralized models. This is reinforced by the high proportion of participants reporting limited confidence in their understanding of PrEP and the near-universal desire for counselling and support services. Together, these findings underscore that pharmacy-based PrEP delivery cannot be limited to drug dispensing alone, but must incorporate structured education, adherence counselling, and possibly peer-support mechanisms [28].

Despite overall positive acceptability, women engaged in transactional sex reported persistent stigma-related concerns, including fears of recognition, disclosure, and negative attitudes from pharmacy staff, which were also reflected in the quantitative findings. Similar concerns have been reported in studies of pharmacy-based PrEP delivery, which show that while pharmacies reduce structural barriers such as distance and waiting time, they do not automatically eliminate stigma, particularly for populations at elevated risk of acquiring HIV infection [14], [29]. Our findings reinforce this evidence by showing that stigma is closely linked to trust in provider competence, confidentiality, and privacy. This suggests that pharmacies may only reduce stigma if services are delivered with trained staff, private consultation spaces, and strong confidentiality safeguards; otherwise, decentralization risks reproducing barriers seen in facility-based HIV care.

Despite enthusiasm, women expressed strong fears of being judged, stigma, and discrimination in pharmacy settings concerns echoed almost universally in the quantitative survey. Qualitative narratives further illustrated how misinformation about PrEP, mistrust of pharmacy staff, and previous negative experiences with medication dispensing could undermine uptake. These concerns indicate that pharmacies may only reduce stigma if staff are appropriately trained and services are delivered discreetly, otherwise, they risk reproducing the same barriers encountered in facility-based care [29].

For ART services, two thirds of PLHIV reported willingness to refill medications at community pharmacies, suggesting moderate but not universal readiness for decentralized refill models. Men and individuals previously aware of pharmacy-based ART services were significantly more willing, highlighting the importance of sensitization and communication in shaping acceptance. Notably, quantitative results showed no association between willingness and access-related factors such as travel time or waiting time, which contrasts with qualitative findings, in which participants strongly emphasized convenience as a key perceived benefit. This difference suggests that while convenience is widely recognized in principle, decisions to use pharmacy-based ART refills may be driven more by concerns about confidentiality, cost, and trust in provider competence than by logistical barriers alone. Similar patterns have been reported in prior studies, which found that although community pharmacy ART models improve access, patient uptake is often constrained by stigma concerns and the perceived need for clinical monitoring and counselling provided in facility-based care [10], [12], [28], [30].

Qualitative findings revealed more ambivalence toward pharmacy-based ART refills than toward PrEP delivery. While participants appreciated pharmacies for emergencies, travel, and flexible hours, hospitals remained the preferred sites for routine HIV care because of trusted provider relationships, comprehensive counselling, psychosocial support, and ongoing clinical monitoring. Similar concerns have been reported in community pharmacy ART models in Nigeria and South Africa, where patients valued convenience but still perceived facilities as offering more comprehensive and specialised HIV care [10], [12], [31]. Studies have also highlighted confidentiality concerns and uncertainty about pharmacist expertise as barriers to uptake of decentralised ART services [28], [30]. However, compared to some implementation studies that demonstrated high retention and patient satisfaction after enrolment into pharmacy ART refill programs [12], our findings reflect perspectives prior to large-scale implementation. This difference may explain the greater ambivalence observed in our study, as participants were reacting to a hypothetical model rather than drawing on direct experience. In addition, in Tanzania, ART is universally provided free of charge in public facilities, which may heighten concerns about potential commodification in private retail settings. This context-specific financing concern appears to shape acceptance more strongly than in settings where pharmacy-based ART models are already integrated within publicly funded systems.

Across stakeholder groups, several cross-cutting implementation challenges were identified, particularly infrastructure limitations, workforce shortages, and gaps in HIV specific training. Similar barriers have been documented in studies of pharmacy-based HIV service delivery in sub-Saharan Africa, where inadequate private space, limited staffing, and insufficient clinical training were found to constrain the quality and confidentiality of HIV care provided in retail settings [13], [31]. Evidence from implemented pharmacy ART models suggests that these challenges can be mitigated through targeted strategies, including formal certification and training programs for pharmacists, integration of standardized dispensing and counselling protocols, and establishment of clear referral linkages with health facilities [10], [12]. Concerns about medication diversion and inappropriate use also align with prior literature highlighting the need for strong regulatory oversight and monitoring systems when decentralizing ART to private sector outlets [23],[31]. Studies further emphasize the importance of interoperable digital health records to support continuity of care, track refills, and prevent duplication or misuse across multiple dispensing sites. Together, these findings suggest that successful scale up of pharmacy-based HIV service delivery will depend less on patient demand alone and more on strengthening regulatory, training, and health system integration mechanisms that ensure safe and accountable implementation.

Importantly, both women engaged in transactional sex and people living with HIV, as well as some pharmacists and policymakers, viewed pharmacies as having the potential to normalize HIV prevention and treatment by embedding these services within routine healthcare encounters, as described elsewhere [34], [35]. This “integration effect” was perceived as a possible stigma-reduction mechanism, provided confidentiality is preserved and clients retain the option to choose between facilities and pharmacies. Such findings suggest that community pharmacies may function best as complementary refill points for stable clients rather than full substitutes for facility-based HIV care.

## Limitations

This study has several limitations. Expressed willingness to use pharmacy-based PrEP or ART services may not reflect actual uptake once implemented. Findings relied on self-reported data and may be influenced by social desirability bias, particularly for sensitive topics such as HIV status, sex work, and stigma. The quantitative PrEP sample was relatively small, reducing statistical power and generalizability, while qualitative participants were purposively selected and limited in number, especially policymakers and pharmacists, potentially restricting the diversity of perspectives captured. The study was conducted within an urban context, which may limit transferability to other settings, especially rural and semi-urban areas. Finally, the study did not include economic analyses or objective assessments of pharmacy readiness, infrastructure, or staffing, which are critical for informing large-scale implementation.

## Conclusions

This study demonstrates substantial but differentiated acceptability of community pharmacy–based HIV service delivery. Interest was high for PrEP delivery among women engaged in transactional sex, reflecting the perceived benefits of convenience, accessibility, and reduced structural barriers. In contrast, the acceptability of pharmacy-based ART refills among people living with HIV was more moderate. While pharmacies were recognized as convenient for emergencies and travel, many participants preferred to continue routine ART care in health facilities due to trusted provider relationships, counselling support, and concerns about confidentiality and potential costs. Across both groups, acceptability was highly conditional and depended on assured confidentiality, maintenance of free or affordable medication, trained pharmacy personnel, and private consultation spaces. Stakeholders further identified workforce capacity, infrastructure readiness, regulatory oversight, digital health integration, and sustainable financing as key determinants of feasibility. Taken together, these findings suggest that community pharmacies are most likely to function as complementary platforms, particularly for expanding PrEP access and providing ART refills for stable clients, rather than as full substitutes for comprehensive facility-based HIV care. Further implementation research and pilot evaluations are needed to assess real world uptake, cost effectiveness, and long-term clinical outcomes.

## Supplementary Material

This is a list of supplementary files associated with this preprint. Click to download.

- Supplementaryfile1questionnaires.docx
- Supplementaryfile2.docx

## Figures and Tables

**Table 1:** Sociodemographic characteristics of respondents and willingness to receive PrEP through community pharmacy (N = 93).

Variable	Category	Category sample (%)	Willing to received PrEP at pharmacy		p- value*
			Yes, n (%)	No, n (%)	
Age (yrs)	Mean ± SD: 28.5 ± 6.4				
	≤ 22	12 (12.9%)	7 (10.1%)	5 (20.8%)	0.39
	23–27	40 (43%)	30 (43.5%)	10 (41.7%)	
	≥ 28	41 (44.1%)	32 (46.4%)	9 (37.5%)	
Age at initiation of transactional sex (yrs)	Mean ± SD: 25.3 ± 5.4				
	≤ 22	26 (28%)	17 (24.6%)	9 (37.5%)	0.31
	23–27	43 (46.2%)	35 (50.7%)	8 (33.3%)	
	≥ 28	24 (25.8%)	17 (24.6%)	7 (29.2%)	
Duration in sex work (yrs)	Median (IQR): 2 (1–4)				
	< 2	37 (39.8%)	27 (39.1%)	10 (41.7%)	1
	2–5	41 (44.1%)	31 (44.9%)	10 (41.7%)	
	≥ 6	15 (16.1%)	11 (15.9%)	4 (16.7%)	
Average clients per day	Median (IQR): 3 (2–5)				
	< 3	40 (43%)	33 (47.8%)	7 (29.2%)	0.11
	≥ 3	53 (57%)	36 (52.2%)	17 (70.8%)	
Marital status	Married	4 (4.3%)	4 (5.8%)	0 (0%)	0.23
	Not married	89 (95.7%)	65 (94.2%)	24 (100%)	
Have children	Yes	53 (57%)	41 (59.4%)	12 (50%)	0.42
	No	40 (43%)	28 (40.6%)	12 (50%)	
Employment status	Employed	25 (26.9%)	16 (23.2%)	9 (37.5%)	0.17
	Not employed	68 (73.1%)	53 (76.8%)	15 (62.5%)	
Education level	No formal education	2 (2.2%)	2 (2.9%)	0 (0%)	0.05
	Primary	23 (24.7%)	15 (21.7%)	8 (33.3%)	
	Secondary	48 (51.6%)	37 (53.6%)	11 (45.8%)	
	Diploma	6 (6.5%)	2 (2.9%)	4 (16.7%)	
	Degree or higher	14 (15.1%)	13 (18.8%)	1 (4.2%)	
Primary work location	Street-based	21 (22.6%)	16 (23.2%)	5 (20.8%)	0.99
	Brothel	6 (6.5%)	4 (5.8%)	2 (8.3%)	
	Bar/club	30 (32.3%)	22 (31.9%)	8 (33.3%)	
	Hotel/lodge	20 (21.5%)	15 (21.7%)	5 (20.8%)	
	**Others	16 (17.2%)	12 (17.4%)	4 (16.7%)	
Consistent condom use	Always	63 (67.7%)	45 (65.2%)	18 (75%)	0.38
	Not always	30 (32.3%)	24 (34.8%)	6 (25%)	
Experienced violence / coercion	Yes	51 (54.8%)	40 (58%)	11 (45.8%)	0.3
	No	42 (45.2%)	29 (42%)	13 (54.2%)	
Other sources of income apart from sex work	No	59 (63.4)	46 (66.7%)	13 (54.2%)	0.27
	Yes	34 (36.6)	23 (33.3%)	11 (45.8%)	
Condom use	Always	63 (67.7)	45 (65.2%)	18 (75%)	0.38
	Not always	30 (32.3)	24 (34.7%)	6 (25%)	
Time since last HIV test	≤ 2 months	84 (90.3)	61 (88.4%)	23 (95.8%)	0.28
	> 2 months	9 (9.7)	8 (11.6%)	1 (4.2%)	
Ever heard of PrEP	No	23 (24.7)	19 (27.5%)	4 (16.7%)	0.29
	Yes	70 (75.3)	50 (72.5%)	20 (83.3%)	

Key:

* Chi-square test of association computed for willingness to obtain pre-exposure prophylaxis (PrEP) at pharmacy

* Others (e.g., online-based services, private residences, escort agencies, massage parlous or saunas, clients’ homes, and other informal venues

**Table 2:** Participant preferences and concerns about PrEP services through community pharmacy (N = 93).

Variable	Category	n (%)
Preferred source of pre-exposure prophylaxis (PrEP) information	Health worker	56 (60.2)
	*Others	37 (39.8)
Confidence in understanding PrEP	Confident	30 (32.3)
	Not confident	63 (67.7)
Preferred PrEP facility	Pharmacy	58 (62.4)
	Non-pharmacy	35 (37.6)
Preferred provider sex	Male health worker	10 (10.8)
	No preference	83 (89.2)
Preferred refill frequency	Monthly	18 (19.4)
	≥ 2 months	75 (80.6)
Interest in long-acting injectable PrEP	Yes	72 (77.4)
	No	21 (22.6)
Peer support for PrEP	Yes	42 (45.2)
	No	51 (54.8)
Preference for counselling/support	Yes	92 (98.9)
	No	1 (1.1)
**What are the concerns about PrEP services through community pharmacy?		
More privacy		22 (23.7)
Shorter waiting time		1 (1.1)
Convenient location		47 (50.5)
Fear of being judged		92 (98.9)
Lack of trust in pharmacist		24 (25.8)
Medication quality concerns		13 (14)
Stockouts		6 (6.5)
Cost		54 (58.1)
Fear of stigma/ discrimination		46 (49.5)

Key:

PrEP: pre-exposure prophylaxis

* Others (including social media platforms, peers/friends, and community outreach worker

** Participant was given an option of tick all apply

**Table 3:** Participant characteristics and the willingness to refill ART at a community pharmacy (N = 341).

Variable	Category	n (%)	Willingness to refill ART at pharmacy		*p*- value*
			Yes n (%)	No n (%)	
**Sex**	Male	84 (24.6%)	66(29.3%)	18(15.5%)	0.1
	Female	257 (75.4%)	159(70.7%)	98(84.4%)	
**Age (years)**	Median (IQR): 40 (32–47)				
	< 18	2 (0.6%)	2 (0.9%)	—	0.46
	18–24	29 (8.5%)	20 (8.9%)	9 (7.8%)	
	25–34	75 (22%)	48 (21.3%)	27(23.3%)	
	35–44	110 (32.3%)	71 (31.6%)	39(33.6%)	
	45–54	82 (24%)	57 (25.3%)	25(21.6%)	
	≥ 55	41 (12%)	27 (12%)	14 (12%)	
**Time since HIV diagnosis (years)**	Median (IQR): 6 (4–10)				
	< 2 years	18 (5.3%)	12 (5.3%)	6 (5.2%)	0.50
	2–5 years	129 (37.8%)	90 (40%)	39(33.6%)	
	> 5 years	194 (56.9%)	123(54.7%)	71(61.2%)	
**Marital status**	Single	84 (24.6%)	58 (25.8%)	26(22.4%)	0.23
	Married	179 (52.5%)	121(53.8%)	58(50%)	
	Divorced/Separated	53 (15.5%)	34 (15.1%)	19(16.4%)	
	Widowed	25 (7.3%)	12 (5.3%)	13(11.2%)	
**Have children**	Yes	293 (85.9%)	197(87.6%)	96(82.8%)	0.23
	No	48 (14.1%)	28 (12.4%)	20(17.2%)	
**Employment status**	Employed	74 (21.7%)	55 (24.4%)	19(16.4%)	0.20
	Not employed	72 (21.1%)	44 (19.6%)	28(24.1%)	
	Self-employed	195 (57.2%)	126 (56%)	69(59.5%)	
**Highest education level**	No formal education	7 (2.1%)	4 (1.8%)	3 (2.6%)	0.94
	Primary	186 (54.5%)	121(53.8%)	65(56%)	
	Secondary	121 (35.5%)	82 (36.4%)	39(33.6%)	
	Diploma	17 (5%)	12 (5.3%)	5 (4.3%)	
	Degree or higher	10 (2.9%)	6 (2.7%)	4 (3.4%)	
**Current ARV refill location**	Public facility	164 (48.1%)	102(45.3%)	62(53.4%)	0.16
	Private facility	177 (51.9%)	123(54.7%)	54(46.6%)	
**Time to reach clinic (minutes)**	Median (IQR): 45 (30–60)				
	< 30	52 (15.2%)	28(12.4%)	24(%)	0.13
	30–60	229 (67.2%)	157(69.8%)	72(%)	
	≥ 60	60 (17.6%)	40(17.8%)	20(%)	
**Time spent at clinic (minutes)**	Median (IQR): 30 (20–45)				
	< 120	306 (89.7%)	201(89.3%)	105(90.5%)	0.73
	≥ 120	35 (10.3%)	24(10.7%)	11(9.5%)	
**Medication reminder/support**	Self	266 (78%)	175(77.8%)	91(78.4%)	0.91
	Partner	29 (8.5%)	18(8%)	11(9.5%)	
	Family	34 (10%)	24(10.7%)	10(8.6%)	
	Peer/Support group	12 (3.5%)	8 (3.6%)	4 (3.4%)	
**Ever missed ART refill due to clinic challenges**	Yes	7 (2.1%)	6 (2.7%)	1(0.9%)	0.27
	No	334 (97.9%)	219 (97.3%)	115 (99.1%)	

Key:

ART: Antiretroviral therapy

IQR: Interquartile range

* : Chi-square test

**Table 4:** Awareness, experience, perceived benefits, concerns, and preferences regarding community pharmacy–based ART services (N = 341).

Variable	Category / response	n (%)
**Awareness and experience**		
Heard of pharmacy-based ART services	Yes	50 (14.7%)
Ever received ART from a private pharmacy	Yes	0 (0.0%)
* **Perceived benefits of receiving ART from a community pharmacy**		
Greater privacy/confidentiality	Selected	14 (4.1%)
Close to home	Selected	180 (52.8%)
Shorter waiting time	Selected	217 (63.6%)
Longer/flexible opening hours	Selected	83 (24.3%)
Reduced transportation costs	Selected	214 (62.8%)
Convenience	Selected	15 (4.4%)
Friendly staff	Selected	3 (0.9%)
* **Concerns and barriers regarding pharmacy-based ART**		
Fear of stigma or being identified	Selected	255 (74.8%)
High cost of services or medicines	Selected	78 (22.9%)
Drug stock-outs / unreliable supply	Selected	43 (12.6%)
Concerns about drug quality	Selected	79 (23.2%)
Pharmacist lacks HIV expertise	Selected	24 (7.0%)
Confidentiality not assured	Selected	209 (61.3%)
Poor record-keeping	Selected	4 (1.2%)
Would accept long-acting injectable ART (every six month)?	Yes	313 (91.8%)
	No	28 (8.2%)
* **Other services desired at the pharmacy**		
HIV testing and counseling		70 (20.5%)
Treatment adherence counseling		197 (57.8%)
Sexual and reproductive health services		112 (32.8%)
Sexually transmitted infection testing		122 (35.8%)
General health screening		207 (60.7%)

* Participant was requested to tick all apply

**Table 5:** Perceived acceptability of community pharmacy–based ART refills among people living with HIV

Theme	Sub-themes	Representative quotes
**Perceived convenience and proximity of pharmacies**	• Shorter waiting times • Easier geographic access • Avoidance of hospital queues	*“It would be good, because many people fear hospitals because of the long processes and queues. Someone asks themselves, ‘Should I go and line up?’ and ends up not going. Pharmacies, however, are much easier to reach for people who avoid clinics.” — IDI 01, Female, 27 years* *“You cannot walk two streets without finding a pharmacy, but hospitals are far. If services move closer to people, many more women will be reached.”* — *IDI 07, Female, 27 years*
**Privacy and reduced stigma in pharmacy settings**	• Fear of being labelled at hospitals • Discreet encounters • Avoidance of HIV-specific clinics	*“In government hospitals the HIV section is known. If you go there just for prevention, people already start thinking you are infected. At pharmacies you feel more private and less watched.”* — *IDI 03, Female, 34 years* *“Going to hospital makes me afraid that people will think I am taking ARVs, but at the pharmacy it feels normal—you just pick medicine and leave.”* — *IDI 06, Female, 30 years*
**Affordability and comfort compared with hospitals**	• Lower perceived costs • Flexible spending • Feeling physically and socially comfortable	*“Hospitals are expensive because you have to pay to see a doctor, but at pharmacies even with a small amount of money you can still go and buy medicine.”* — *IDI 04, Female, 23 years* *“If PrEP comes to shops around us, it becomes easier and increases confidence, because people fear HIV clinics. In pharmacies no one questions you about what the medicine is for.”* — *IDI 05, Female, 22 years*
**Need for education and sensitization**	• Training for sex workers • Counselling skills • Awareness campaigns	*“Education is the biggest issue. You must hold seminars and explain to sex workers clearly, because without understanding people become afraid to use these medicines.”* — *IDI 01, Female, 27 years* *“Pharmacies should also have trained people who can explain these drugs properly, just as you explained to me today, so women know what they are taking.”* — *IDI 06, Female, 30 years*
**Concerns about pharmacy staff competence**	• Risk of wrong medication • Limited HIV-specific knowledge • Loss of trust	*“When you talk about pharmacies, they must first be given education. If they are trained well, they will understand these drugs better. Without that training, clients will not trust them to give correct advice.” — IDI 07, Female, 27 years* *“I once went for ear pain and was given drugs meant for menstrual cramps. Another pharmacy corrected it, but that experience made me fear what damage the first medicines might have caused.”* — *IDI 03, Female, 34 years*
**Fear and misconceptions about PrEP**	• Rumors about weakened immunity • Association with HIV infection • Internalized stigma	*“People fail to use PrEP because taking daily tablets feels like accepting that you are infected. Many have not accepted themselves and fear being judged.”* — *IDI 07, Female, 27 years* *“Some people told me that PrEP weakens your immunity, and because of that advice I hesitated to start using it.”* — *IDI 01, Female, 27 years*

Key:

IDI: Participant who participated in in-depth interview plus the identification number assigned during an interview

PrEP: Pre-exposure prophylaxis

**Table 6:** Facilitators, barriers, and conditions for implementing ART refills at community pharmacies from in-depth and key informant interviews

Theme	Sub-themes	Representative quotes
Perceived convenience of community pharmacies	• Reduced travel distance • Avoiding long clinic queues • Flexible opening hours	*“When we hear about collecting medicines from pharmacies, we feel relieved because it would reduce the long queues at hospitals. Sometimes you arrive at noon, and you are told the fingerprint machine is not working or the network is down, and you end up staying there for hours. Even if you came early, you still leave late. That is why when we hear about pharmacies, we feel happy.”* — *FGD17*, *Female, 33 years* *“I could even go late at night because some pharmacies operate 24 hours. That would really help me, because I plan my time around my small business. At hospitals, if you arrive late you might be punished by being told to come back the next day, but at pharmacies I feel I could go at a time that suits me.”* — *FGD13*, *Female, 35 years*
Use of pharmacies for emergencies or travel	• Sudden travel • Distance from health facilities	*“If a funeral suddenly happens and I must travel the same night, and the health facility is far away, I cannot manage to go back to the clinic. I would just go to a pharmacy, get my medicines, and continue with the journey instead of missing my dose.”* — *FGD08*, *Female, 25 years*
Fear of stigma and breach of confidentiality	• Being recognized by neighbors • Gossip • Distrust of pharmacy staff	*“Honestly, I am afraid. My fear comes from secrecy and stigma. Pharmacies are in the same neighborhood where we live. If you meet a neighbor’s child there, that child might go and tell their mother, and the story spreads from one person to another. Even if nobody says anything directly, you keep worrying that people already know about you.”*— *FGD14*, *Female, 28 years* *“The pharmacy is different from the hospital because it is surrounded by the community. You might go there and ask for medicine, and people sit watching you. The attendants are young nurses who are still learning, and you fear they might start talking about you. For me, I completely disagree with using pharmacies for this reason.”* — *FGD18*, *Female, 41 years*
Concern about potential user fees	• Pharmacies seen as businesses • Fear ART will no longer be free	*“Once medicines start being provided in pharmacies it becomes business. You cannot go there without money. Even Panadol is not given for free, so what about these tablets? At hospitals we get them free, but in pharmacies they will start selling them, and that will really affect us.”* — *FGD15*, *Female, 45 years* *“These drugs are currently free at health facilities, but when you talk about pharmacies it means trade will start. Someone must make profit. That means we who are used to free treatment will face serious challenges.”* — *FGD12*, *Male, 31 years*
Trust in hospital-based care	• Counselling and peer support • Familiar providers • Clinical monitoring	*“At the hospital there are many services. We get weighed, tested, counselled, and we meet other patients who encourage each other. The nurses there already know us.* *In pharmacies I would just go, pick the drugs, and leave while feeling afraid, but at hospital I feel free.” — FGD11, Female, 40 years*
Concerns about provider competence in pharmacies	• HIV-specific training • Ability to manage side-effects • Quality of follow-up	*“I have changed treatment more than five times because of side-effects. When I went to a doctor who knew my history, he changed my regimen and the problem stopped. Now I worry that if I go to a pharmacy, the person there might not understand these issues the way clinicians at the hospital do.”* — *FGD13*, *Female, 35 years*
Conditional willingness to use pharmacies	• Free ART • Confidentiality safeguards • Trained staff	*“If the issue of payment was removed and privacy was properly protected, then I could accept going to a pharmacy. But if I still have to pay transport and also buy the medicines, and people can see me there, then it becomes difficult.”* — *FGD12*, *Male, 31 years*
Suggested safeguards for implementation	• Private consultation rooms • Dedicated ART providers • Discreet refill procedures	*“There should be a separate private room. I enter there and meet a nurse who is trained in this service, we talk privately, I show my card, get my medicines, put them in my bag, and leave. No one else should be able to see or hear what is happening inside.”* — *FGD16*, *Male, 38 years*

Key:

ART: Antiretroviral therapy

FGD: Participant who participated in focused group discussion plus the identification number assigned during an interview

**Table 7.** Facilitators, barriers, and conditions for implementing ART refills at community pharmacies from in-depth and key informant interviews

Theme	Sub-themes	Representative Quotes
**Accessibility and public-health value of pharmacy-based ART/PrEP**	• Proximity and timesaving • Reduced congestion at clinics • Demand for PEP/PrEP in communities	*“Nowadays when many people fall sick, instead of going to hospitals they first look for immediate help… so if ARVs or PrEP services were available in our pharmacies, the first benefit would be accessibility—people would be able to get services easily without wasting time.”* — *IDI12, Male, 32 years* *“If these services were available in pharmacies, it would be much easier for anyone… community pharmacies are many compared to health facilities.”* — *IDI13, Female, 38 years*
**Risk of misuse and need for strong dispensing controls**	• Diversion to animals • Fabricated exposure stories • Partial dosing	*“Some people have been using ARVs to feed livestock… a person may come and fabricate a story just to request PrEP… you cannot know how it will be used.”* — *IDI10, Male, 30 years* *“Many people do not complete the dose… they keep the remaining pills or give them to someone else without considering the expiry date.”* — *IDI10, Male, 30 years*
**Regulatory and financing uncertainty**	• Price regulation vs free provision • Government reimbursement • Sustainability beyond pilots	*“No businessperson likes someone to enter their shop and take something for free… there has to be a way for them to benefit…”* — *IDI10, Male, 30 years* *“A client may come for ARVs or PrEP, but they are also customers… they may come for this service and also get other services there in the pharmacy”* — *IDI14, Male, 37 years* *“Government could exempt some of these fees for private pharmacies… so they cover the costs they thought they would use when providing services to HIV clients.”* — *KII01, Male, 45 years*
**Infrastructure limitations for confidential care**	• Lack of private rooms • Open counters • Confidentiality risks	*“If you begin an assessment, you may find that 90% of pharmacies do not even have a private room… the main issue is infrastructure.”* — *IDI11, Male, 31 years* *“Most community pharmacies were not designed to have a special room… ARV services require a high level of confidentiality.”* — *IDI08, Male, 28 years*
**Workforce shortages and workload pressures**	• Few staff per shift • Time-intensive counselling • Business disruption	*“During one shift there is usually only one person… there are ordinary customers and those seeking PEP or ART… that other client needs more time.”* — *IDI13, Female, 30 years* *“An ARV client may take five or ten minutes… that affects business and customer flow.”* — *IDI08, Male, 28 years*
**Training and competency requirements**	• Knowledge gaps • Counselling skills • Documentation	*“You may find that we hire a young person straight from college who has never provided ARV services… they really need adequate training.”* — *IDI12, Male, 32 years* *“Service providers need more training, especially on confidentiality and on giving proper counselling.” – IDI08, Male, 28 years*
**Digital systems and data integration**	• Harmonized software • National interoperability • Reporting systems	*“A patient may be served in Dar es Salaam and then later in Mwanza that pharmacy should be able to see what medicines were collected… there must be a nationwide system.”* — *IDI09, Female, 36 years* *“The systems used in pharmacies must be interoperable; otherwise, confusion will arise in the records.”* — *IDI08, Male, 28 years*
**Client selection, referral and supply chains**	• Stable-patient criteria • Referral mechanisms • Facility-linked supply	*“There must be a proper mechanism… even if a patient goes to another pharmacy the records should be visible so that patients are properly followed up.”* — *IDI09, Female, 36 years*
**Stigma reduction through integrated service delivery**	• Normalizing HIV care • Patient choice • Mixed-service settings	*“Providing these services in pharmacies would reduce waiting times and queues… and people may feel more comfortable than going to specialized HIV clinics.”* — *IDI08, Male, 28 years*

Key:

ART: Antiretroviral therapy

ARVs: Antiretrovirals

IDI: Participant who participated in in-depth interview plus the identification number assigned during an interview

KII: Participant who participated in key-informant interview plus the identification number assigned during an interview

PEP: Post-exposure prophylaxis

PrEP: Pre-exposure prophylaxis

## Abbreviations

ART: Antiretroviral therapy
CP: Community pharmacy
FGD: Focus group discussion
HIV: Human Immunodeficiency Virus
IDI: In-depth interview
KII: Key informant interview
PEP: Post-exposure prophylaxis
PLHIV: People living with HIV
PrEP: Pre-exposure prophylaxis
